# Heterogeneous dynamics in DNA site discrimination by the structurally homologous DNA-binding domains of ETS-family transcription factors

**DOI:** 10.1093/nar/gkv267

**Published:** 2015-03-30

**Authors:** Gaofei He, Ana Tolic, James K. Bashkin, Gregory M. K. Poon

**Affiliations:** 1 Department of Chemistry and Biochemistry and Center for Nanoscience, University of Missouri – St. Louis, St. Louis, MO 63121, USA; 2 College of Pharmacy, Washington State University, Spokane, WA 99210, USA

## Abstract

The ETS family of transcription factors exemplifies current uncertainty in how eukaryotic genetic regulators with overlapping DNA sequence preferences achieve target site specificity. PU.1 and Ets-1 represent archetypes for studying site discrimination by ETS proteins because their DNA-binding domains are the most divergent in sequence, yet they share remarkably superimposable DNA-bound structures. To gain insight into the contrasting thermodynamics and kinetics of DNA recognition by these two proteins, we investigated the structure and dynamics of site discrimination by their DNA-binding domains. Electrophoretic mobilities of complexes formed by the two homologs with circularly permuted binding sites showed significant dynamic differences only for DNA complexes of PU.1. Free solution measurements by dynamic light scattering showed PU.1 to be more dynamic than Ets-1; moreover, dynamic changes are strongly coupled to site discrimination by PU.1, but not Ets-1. Interrogation of the protein/DNA interface by DNA footprinting showed similar accessibility to dimethyl sulfate for PU.1/DNA and Ets-1/DNA complexes, indicating that the dynamics of PU.1/DNA complexes reside primarily outside that interface. An information-based analysis of the two homologs’ binding motifs suggests a role for dynamic coupling in PU.1's ability to enforce a more stringent sequence preference than Ets-1 and its proximal sequence homologs.

## INTRODUCTION

**Figure 1.**
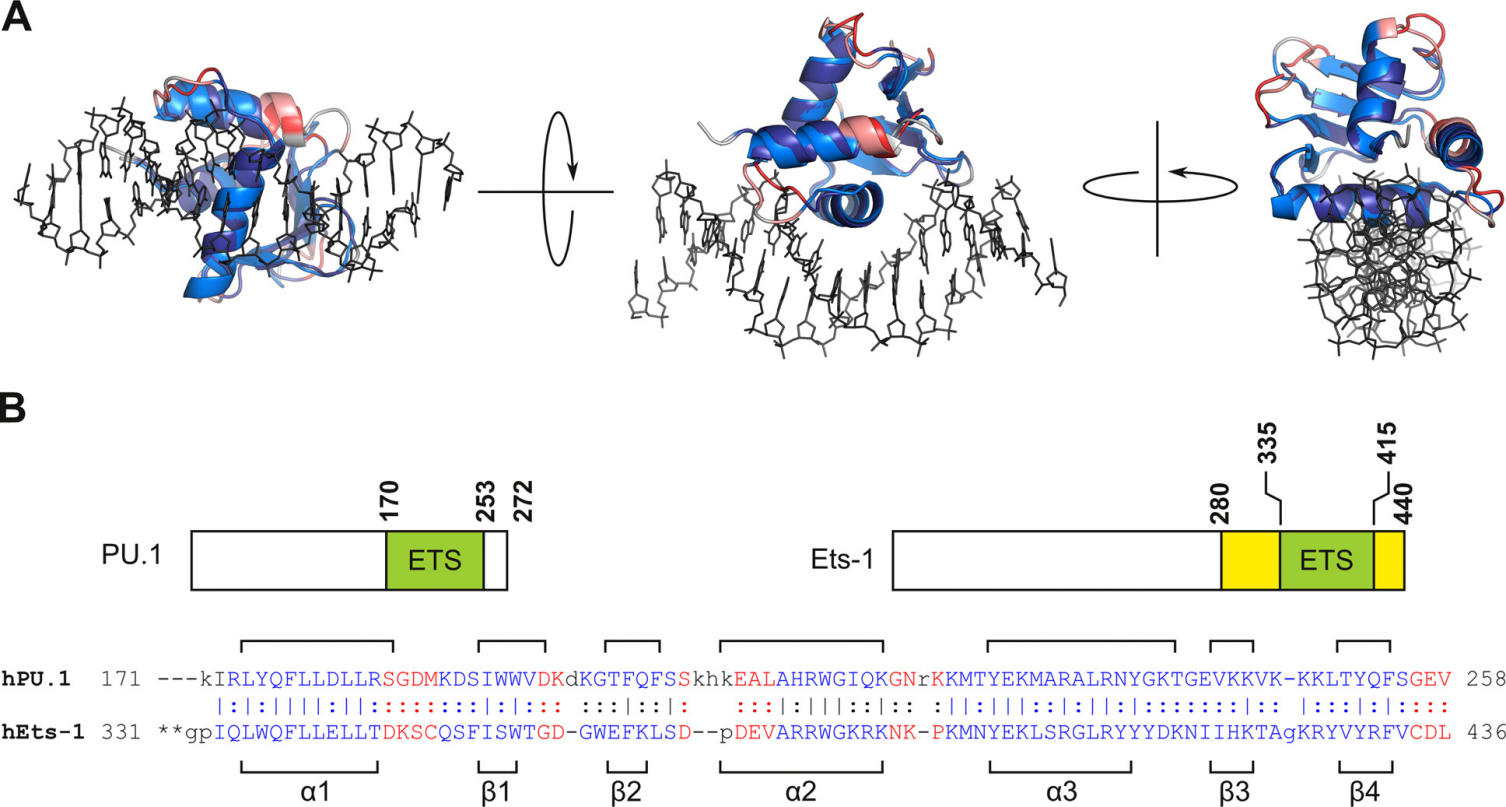
The sequence-divergent ETS domains of PU.1 and Ets-1 map to highly homologous structures. (**A**) Structural alignment of the ETS domains of PU.1 (PDB: 1PUE) and Ets-1 (1K79) in their high-affinity DNA co-crystal structures using the RAPIDO algorithm (13). The two ETS domains were flexibly aligned to optimize overlap of matching sets of secondary structure elements (termed rigid bodies), colored blue and purple for PU.1 and Ets-1. Non-aligned, flexible segments are colored red (PU.1) and magenta (Ets-1). Sticks connect matched C^α^ atoms. The two domains align to a global RMSD of 1.4 Å for C^α^ atoms, well within the resolution of the source structures (1PUE: 2.1 Å; 1K79: 2.4 Å). If only the rigid bodies are considered, the alignment improves to 0.84 Å. The choice of asymmetric units made no meaningful differences in the alignment. DNA (shown only for PU.1) is rendered as lines. (**B**) Domain structure of human PU.1 and Ets-1. Unlike PU.1, the ETS domain of Ets-1 is flanked by helices (yellow) that unfold upon DNA binding and attenuate DNA-binding affinity; the loss of either flanking segment abolishes autoinhibitory effects such that Ets-1ΔN331 behaves as a minimal ETS domain (19). A primary sequence alignment of the ETS domains of the two proteins is also shown, with assigned secondary structure elements in brackets.

Members of the ETS family of transcription factors are diverse in their interactions with target genes and chromatin *in vivo*. For example, the ETS-family member PU.1 is a pioneering transcription factor (1,2): it can bind DNase I-inaccessible chromatin and methylated DNA, initiate nucleosomal remodeling by promoting local histone modifications, and direct other transcription factors by cooperative recruitment (3–7). The capability to resolve nucleosomes is not a class property of ETS proteins, however, as another ETS member, Ets-1, is not a pioneer (8). This and other functional differences reflect the profound variation in the amino acid sequences that encode the eponymous DNA-binding domains of ETS proteins, with PU.1 and Ets-1 representing the extremes of sequence divergence (∼30% homology) (9). Nevertheless, ETS proteins share broadly overlapping DNA site preferences around a 5′-GGA(A/T)-3′ consensus (10) and strong structural homology. The backbone trajectories of PU.1 and Ets-1 with high-affinity DNA (11,12) are superimposable well within the precision of the respective co-crystal structures (Figure 1)(13). Thus, given their distinct functional profiles and divergent primary sequences on the one hand, yet strong structural conservation and overlapping sequence preferences in the other, PU.1 and Ets-1 represent excellent models for understanding how functionally non-redundant transcription factor homologs execute member-specific DNA site recognition.

To better understand the mechanisms of DNA recognition by ETS proteins, we have been studying the thermodynamics and kinetics of DNA binding by the ETS domains of Ets-1 and PU.1 to high- and low-affinity sequence-specific sites. We found that the high- and low-affinity DNA complexes exhibit markedly differentiated hydration and electrostatic properties in the case of PU.1, but not Ets-1 (14,15). These differences are experimentally manifest by way of the complexes’ sensitivity to water and ion activities: whereas high-affinity PU.1/DNA complexes are destabilized by osmotic stress, low-affinity complexes are only very weakly sensitive to the osmotic environment, as are both high- and low-affinity Ets-1/DNA complexes (14,15). Similarly, the DNA complexes of PU.1 are sensitive to monovalent ion concentrations in a markedly sequence-dependent manner, but not for Ets-1/DNA complexes (15). Finally, salt perturbs the kinetics of site recognition by the two proteins in opposite directions (15,16). These sharp contrasts in the coupling of their DNA complexes to the solution environment suggest a profound heterogeneity in the mechanisms of DNA discrimination between the two ETS paralogs and, by implication, within the broader ETS family.

Currently, the structural underpinnings for the differential properties among ETS transcription factors remain elusive. Structures of ETS/DNA complexes are limited mostly to high-affinity species (reviewed in 17), which show universally strong conservation for the DNA-bound proteins. However, high-affinity structures alone cannot fully inform the basis of site discrimination without comparative data on low-affinity complexes, particularly in light of the sequence-dependent properties of PU.1/DNA complexes (14,18). A direct comparison of high- and low-affinity complexes for different ETS proteins is therefore essential for understanding site recognition by ETS transcription factors. Here, we report a comparative characterization of high- and low-affinity DNA complexes formed by the ETS domains of PU.1 and Ets-1 in solution. The data show that DNA site identity unmasks major changes in the dynamics in PU.1/DNA complexes that do not accompany their Ets-1 counterparts, and point to conformational dynamics as a novel differentiator in DNA site discrimination by ETS proteins.

## MATERIALS AND METHODS

### Protein expression and purification

The recombinant ETS domains of murine PU.1 (residues 167–272, termed ΔN167) and Ets-1 (residues 311–440, termed ΔN311, denoting the minimal ETS domain (19); and residues 280–440, a gift from Dr Lawrence P. McIntosh termed ΔN280, denoting the autoinhibited form) were expressed and purified as previously described (14,15). Briefly, BL21*(DE3) *Escherichia coli* harboring the appropriate plasmid was grown to OD_600_ ∼0.6 and induced with 0.5 mM Isopropyl β-d-1-thiogalactopyranoside (IPTG) at 30°C for ∼4 h. After purification on Co-NTA, thrombin cleavage, and size-exclusion chromatography, protein was eluted in 10 mM Tris–HCl (pH 7.4) containing 0.5 M NaCl and (for Ets-1 constructs) 0.5 mM Tris(2-carboxyethyl)phosphine hydrochloride (TCEP). Protein concentrations were measured spectrophotometrically at 280 nm using the following extinction coefficients: 22 460, 32 430 and 39 880 M^−1^ cm^−1^ for PU.1ΔN167, Ets-1ΔN331 and Ets-1ΔN280.

### DNA constructs

The high- and low-affinity sites used for PU.1 are 5′-AGC**GGAA**GTG-3′ and 5′-AAA**GGAA**TGG-3′ (consensus in bold) (20). The sites used for Ets-1 are GCC**GGAA**GTG (termed SC1, high-affinity) and TCC**GGAA**ACC (SC12, low-affinity) (21). ETS binding sites were assembled from synthetic oligonucleotides at ∼0.5 mM duplex, and their concentrations determined spectrophotometrically at 260 nm using nearest-neighbor methods (22).

### DNA circular permutation

As detailed in **SM1** of *Supplementary Methods*, pBend5-based plasmids (23) were digested with specific restriction endonucleases to yield eleven distinct fragments of constant length (143 bp) in which an ETS binding site occurs at defined intervals along the fragment. Each fragment was end-labeled with [γ-^32^P]-ATP using T4 polynucleotide kinase and purified by agarose gel electrophoresis. The fragments were incubated to equilibrium with sub-saturating amounts of their target ETS domain (between 0.1 and 10 μM) and resolved in 12% non-denaturing polyacrylamide gels (5% C, 1× Tris-borate-EDTA (TBE)) at 20 V/cm for 3 h. Gels were digitized by phosphorimagery using a Storm 860 instrument (GE Healthcare). Quantitative analysis of the electrophoretic data to determine the mobilities of the bound and unbound DNA, as well as model-dependent parameter estimation and statistical inference, are detailed extensively in SM2 and SM3 of *Supplementary Methods*.

### Dynamic light scattering (DLS)

ETS domain (200 μM) was extensively dialyzed against phosphate-buffered saline alone or mixed with duplex oligo DNA at a 1:1 molar ratio, filtered (0.45 μm), and measured at 25°C with a Zetasizer Nano ZS instrument (Malvern). For each sample, back-scattering at 173° was integrated for 4 h to ensure overall signal convergence to within 1%, analyzed using the Stokes–Einstein equation, and fitted to log-normal distributions.

### DNA footprinting

DNA fragments (206 bp) harboring ETS binding sites were PCR-amplified from pUC19 plasmids as described (14), except the forward and reverse primers were fluorescently end-labeled at the 5′ end with Alexa Fluor 488 and 6-HEX for capillary electrophoresis (24,25). Gel-purified amplicons were incubated to equilibrium with saturating concentrations (up to 10 μM) of ETS domains in 10 mM Tris–HCl (pH 7.4) containing 150 mM NaCl, 0.1 mM ethylenediaminetetraacetic acid (EDTA), 0.5 g/l bovine serum albumin (BSA) and 0.1 g/l salmon sperm DNA in a final volume of 50 μl. For dimethyl sulfate (DMS) footprinting, 0.25 μl of neat DMS was thoroughly mixed into the sample for 30 s, then quenched with 150 μl of a guanidine thiocyanate quench buffer. For DNase I footprinting, MgCl_2_ was added to 2.5 mM immediately before 1 U of DNase I, and quenched after 30 s as above. Samples were purified with spin columns (Thermo Scientific). For DMS-treated samples, purified DNA was eluted in 10% (v/v) piperidine, heated at 90°C for 5 min, and ethanol-precipitated with 20 μg of glycogen. The pellets were dissolved in water and re-purified with spin columns. For all samples, the final elution volume was 10 μl in TE. Capillary electrophoresis was performed by the University of Missouri DNA Core Facility with an ABI 3730xl DNA Analyzer. Peaks were indexed with GeneMarker software (version 1.97, Softgenetics) (26) and numerically integrated as previously described (14,27).

### Statistical procedures

Statistics and least-square model fitting were performed using Origin (version 9.1, OriginLab). Hypothesis testing for differences between means was performed by *t* tests with adjustment for multiple comparisons to control the false discovery rate (28). Fitted estimates of parameters are given with 95% joint confidence limits and inferences on goodness-of-fit to datasets were performed by Fisher's *F* tests on residual sums of squares.

## RESULTS AND DISCUSSION

### Circular permutation of sequence-specific ETS binding sites reveals distinct structures of PU.1/DNA and Ets-1/DNA complexes

In reported structures of site-specific ETS/DNA complexes, the protein contacts and neutralizes phosphates on one side of the DNA backbone, leading to asymmetric collapse of the helix (29). Our solution studies have revealed significant heterogeneity in counter-ion release upon site binding by PU.1 and Ets-1 (15): whereas Ets-1 binding affinities to high- and low-affinity sites respond identically to bulk salt concentration, in quantitative agreement with the number of phosphate contacts, the corresponding affinities for PU.1 are salt-sensitive in a markedly site-dependent manner. Therefore, we were initially interested in whether the sequence preferences of these ETS homologs might be related to their induction of DNA curvature.

**Figure 2.**
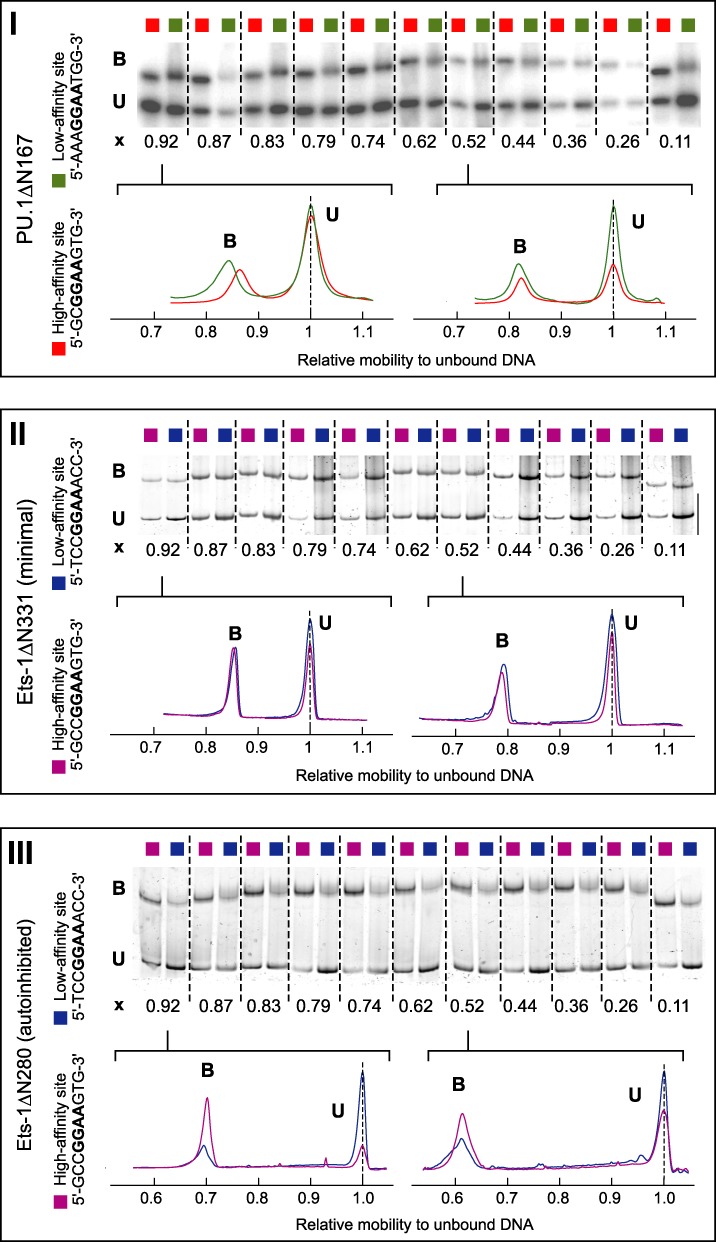
DNA circular permutation reveals structurally distinct site-specific complexes formed by the homologous ETS domains of PU.1 and Ets-1. The high- and low-affinity complexes formed by PU.1ΔN167 (**I**), Ets-1ΔN331 (**II**; minimal ETS domain), and Ets-1ΔN280 (**III**; autoinhibited ETS domain) were probed by polyacrylamide electrophoresis (representative data shown) using circularly permuted DNA fragments as detailed in SM1 of *Supplementary Methods*. Flexure displacement (*x*), the position of the center of the ETS binding sites relative to the length of the entire fragment, is shown for each ETS/DNA complex. To illustrate the relative mobilities of the fragments bound by each protein, intensity traces of two fragments with terminal (*x* = 0.92) and centered (*x* = 0.52) binding sites are shown, offset slightly along the abscissa to align the unbound bands. Quantitation of relative mobilities is detailed in SM2, *Supplementary Methods*.

To probe the curvature of ETS/DNA complexes, we measured the electrophoretic mobilities of circularly permutated ETS binding sites that have been fractionally bound by the ETS domain of PU.1 or Ets-1 (Figure 2). We generated a series of eleven 143-bp DNA fragments that harbor a single 10-bp ETS binding site ranging from one end to the other (SM1, *Supplementary Methods*), and examined site-specific complexes formed by PU.1 and Ets-1 with the same set of high- and low-affinity sites whose thermodynamics and kinetics we have recently reported (15). Localized DNA curvature induced by protein binding led to position-dependent mobilities for the complex (relative to the unbound fragment) that were highest for fragments with terminal binding sites, and lowest for fragments with centered sites. Samples were resolved in gels prepared from the same batch of acrylamide and buffer solutions to eliminate gel-to-gel variation in the electrophoretic matrix. Under these conditions, the standard errors in relative mobility measurements from quadruplicate experiments are ±0.005 or better (Supplementary Table S1), similar to the analytical resolution of the procedure used to quantitate the mobilities (SM2, *Supplementary Methods*).

Differences in relative mobility between corresponding high- and low-affinity complexes of a given ETS domain, at each flexure-to-end distance (flexure displacement, *x*), were inferred by *t* tests with adjustment for multiple comparisons (Supplementary Table S1) (28). Under identical conditions, the relative mobilities of high- and low-affinity sequence-specific complexes formed by the ETS domain of PU.1 (PU.1ΔN167; Figure 2, Panel I) varied systematically in a position-dependent manner. Specifically, the low-affinity PU.1/DNA complex migrated with progressively lower mobility than the high-affinity complex for binding sites situated increasingly nearer the ends of the DNA fragments (i.e. *x* approaching 0 and 1). PU.1 complexes with binding sites near the center of the DNA (*x* near 0.5) showed negligible differences in mobility, regardless of high- or low-affinity binding. For the minimal ETS domain of Ets-1 (Ets-1ΔN331; Figure 2, Panel II), no mobility differences were observed between the high- and low-affinity complexes, after statistical adjustment for multiple testing (Supplementary Table S1). Unlike PU.1, the ETS domain in Ets-1 is flanked by autoinhibitory helices (cf. Figure 1B) that attenuate DNA binding affinity. Regardless of site location, the corresponding high- and low-affinity complexes formed by autoinhibited Ets-1 (Ets-1ΔN280; Figure 2, Panel III) showed indistinguishable mobilities from each other. These qualitatively different results for the three ETS domains argue strongly against their origin in artefacts of electrophoresis. In particular, the mobility differences cannot be attributed to dissociation of low-affinity complexes during electrophoresis. Dissociation of low-affinity complexes would cause mobility to increase and approach that of unbound DNA, but many low-affinity complexes exhibit similar mobilities as their high-affinity counterparts. Therefore, the relative mobilities represent intrinsic differences in the electrophoretic properties among the ETS/DNA complexes.

**Figure 3.**
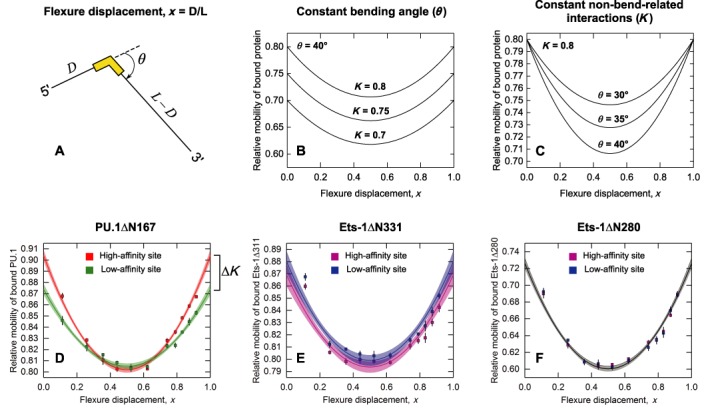
Model-dependent analysis of PU.1/DNA and Ets-1/DNA electrophoretic mobilities suggests a dynamic component in DNA site discrimination by ETS proteins. (**A**) The geometric model used showing the definitions of the bending angle (*θ*) and flexure displacement (*x*). The parabolic relationship between relative mobility and *x* is detailed in (30) and SM3 in *Supplementary Methods*. The model's parameterization of site bending (*θ*) and non-bend-related interactions (*K*) enables a direct assessment of the two factors from the differential mobilities of high- and low-affinity complexes formed by the same ETS protein. In particular, the functional value at *x* = 1 or 0 is equal to *K* regardless of *θ*. (**B**) If *θ* is constant, the model predicts a vertical shift with no change in curvature. (**C**) If *K* is constant, the model predicts a position-dependent difference in relative mobility that is maximal for centered binding sites but vanishes for terminal sites. Unconstrained least-square fits of the model to the data for PU.1ΔN167 (**D**), Ets-1ΔN331 (**E**) and Ets-1ΔN280 (**F**) with 95% confidence bands. Symbols represent mean ± SE of quadruplicate experiments (Supplementary Table S1). Parametric estimates and statistics are given in Supplementary Table S2. For Ets-1ΔN280, a global fit to both datasets is shown.

To interpret the contrasting results between PU.1 and Ets-1 more mechanistically, we analyzed the mobility data with a quantitative model (30) based on the Lumpkin–Zimm reptation theory (31,32). The model considers protein-induced bending as a fixed point-kink (Figure 3A and SM3, *Supplementary Methods*), an approximation appropriate for our constructs in which the binding site spans only 7% of the fragment length. For each ETS/DNA complex, the model fits relative mobility as a quadratic function of flexure displacement, *x*; mobility is minimized at the fragment's midpoint (*x* = 0.5) where flexure causes the greatest deviation from linearity. The key advantage of this model is its ability to separate effects on mobility due to site bending (angle *θ*) and non-bend-related interactions with the gel matrix (*K*). This parameterization is therefore well-suited to quantifying the degree of bending as well as testing if additional interactions contribute to the differential mobilities of high- and low-affinity complexes formed by the same ETS protein. On the one hand, if complexes differ only in their interactions with the gel matrix and are identical in bending angle, a uniform shift in mobilities results at all flexure displacements (Figure 3B). On the other hand, if complexes differ exclusively by a difference in DNA bending, the dispersion in their mobilities would be manifest in fragments with centered sites, and vanish in fragments with terminal sites (Figure 3C).

When applied to the high- and low-affinity DNA complexes of PU.1ΔN167, the model clearly distinguishes the two complexes (*P* < 0.00001, *F* test on residual sum of squares; Supplementary Table S2). Differences in both bend- and non-bend-related effects account for the divergent electrophoretic mobilities as *x* approaches 0 and 1 (Figure 3D). In contrast, the model does not distinguish complexes formed by the minimal (Ets-1ΔN331, *P* = 0.15; Figure 3E) or the auto-inhibited (Ets-1ΔN280, *P* = 0.98; Figure 3F) ETS domain of Ets-1. Importantly, *R*_f_(*x* = 0) = *R*_f_(*x* = 1) = *K* independently of the bending angle (SM3, Supplementary Data); the functional value at *x* = 0 and *x* = 1 therefore provides a direct assessment of the non-bend-related properties from the fitted mobility data. While the difference in non-bend-related effects between the high- and low-affinity complexes of PU.1ΔN167 are well beyond experimental uncertainty (cf. the 95% confidence bands in Figure 3D and Supplementary Table S2), those of either Ets-1 variant are not. In summary, circular permutation discerns non-bend-related determinants of structural heterogeneity among sequence-specific complexes of PU.1 and Ets-1.

### Site-specific changes in conformational dynamics are strongly coupled to DNA binding by the ETS domain of PU.1, but not Ets-1

In the reptation model (SM3, *Supplemental Data*), the dimensionless non-bend parameter *K* describes the electrostatic and frictional properties of the protein-bound (b) and unbound (u) DNA (30,31):

(1) \begin{equation*} K = \frac{{Q_{\rm b} }}{{\xi _{\rm b} }}\frac{{\xi _{\rm u} }}{{Q_{\rm u} }} \approx \frac{{\xi _{\rm u} }}{{\xi _{\rm b} }}, \end{equation*}

where *Q* and *ξ* represent the total effective charge and frictional constant. For complexes formed by the same protein and DNA fragments of fixed length, *Q*_b_ and *Q*_u_ are constant, and given the 2 × 144 = 288 total phosphates in our duplex DNA fragments, nearly identical. Reptation analysis therefore implies interactions that frictionally couple with the polyacrylamide gel matrix contribute differentially to PU.1-bound, but not Ets1-bound complexes, in a site-dependent manner. One mechanism for such frictional coupling would be via conformational dynamics. To examine this notion further, we probed the two proteins in solution (with no gel), with and without DNA duplexes harboring the same high- and low-affinity binding sites introduced above, by dynamic light scattering (DLS).

**Figure 4.**
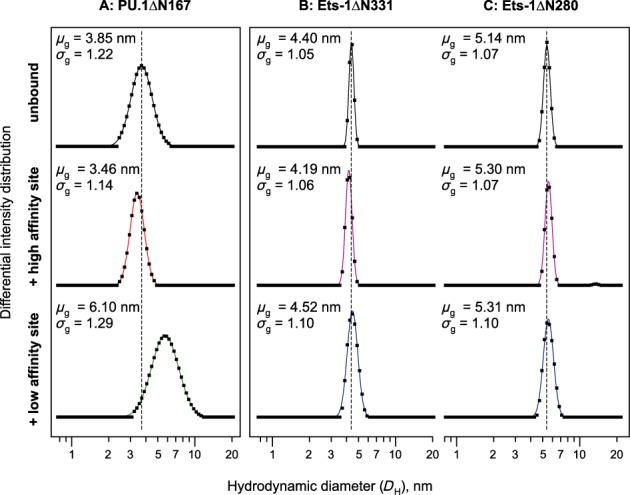
Heterogeneity in conformational dynamics of the homologous ETS domains of PU.1 and Ets-1 in unbound and DNA-bound states. Hydrodynamic diameter (*D*_H_) distributions of (**A**) PU.1ΔN167, (**B**) minimal Ets-1ΔN33, and (**C**) autoinhibited Ets-1ΔN280 in free solution with or without sequence-specific sites as measured by dynamic light scattering. The observed intensity distributions (symbols; integrated over 4 h) were fitted with log-normal distributions (lines). The least-square estimates for median hydrodynamic diameter (geometric mean, *μ*_g_) and dimensionless geometric standard deviation (*σ*_g_) are shown for each species; the standard error in each parameter is ±0.01 unit or lower. A range for *D*_H_ is obtained by multiplying and dividing *μ*_g_ by *σ*_g_. For each protein, the peak (modal) diameter for the unbound species is marked by a dashed line to guide the eye for comparison with the bound states.

DLS analysis for the unbound proteins showed similar median hydrodynamic diameters for PU.1ΔN167 and the minimal ETS domain of Ets-1 (Ets-1ΔN331), but significantly smaller than the autoinhibited Ets-1ΔN280 (Figure 4). However, PU.1ΔN167 exhibits a significantly broader size distribution than both Ets-1ΔN331 and Ets-1ΔN280. The broader PU.1 distribution is not due to polydispersity arising from impurities in the preparation of PU.1ΔN167, as evidenced by sodium dodecyl sulphate-polyacrylamide gel electrophoresis (SDS-PAGE) analysis of the purified protein (Supplementary Figure S1). The data therefore indicate a significantly broader ensemble of structures in PU.1 than Ets-1 at the μs or longer timescale to which the optical mixing system of our DLS instrument is sensitive. This result is consistent with a previous solution NMR study of PU.1 showing significant relative motion between secondary structure elements in the same time régime (33).

To quantitatively generate 1:1 ETS/DNA complexes (27), site-specific duplex oligos were mixed at equimolar concentrations to form 100 μM complexes. High-affinity DNA caused slight downward shifts of the median hydrodynamic diameter for PU.1ΔN167 and Ets-1ΔN331, as well as a significant tightening of the distribution in the case of PU.1ΔN167 only. Conversely, a 1:1 molar mixture with low-affinity DNA increased the median hydrodynamic diameter for Ets-1ΔN331 and significantly more so for PU.1ΔN167, in addition to a broadening of the latter's size distribution. Low-affinity DNA binding may therefore trigger partial melting of folded elements in PU.1, but not Ets-1, causing increased fluctuations in the former. Both high- and low-affinity DNA complexes of Ets-1ΔN280 exhibit right-shifted and broadened size distributions relative to the unbound protein, reflecting the unfolding of the auto-inhibitory helices upon DNA binding (34). The qualitatively varied scattering by the three ETS constructs indicate that they reflect intrinsic dynamic heterogeneity in site binding, rather than simple contributions from the added DNA, which shows essentially identical profiles in the unbound state (Supplementary Figure S2).

In summary, DLS supports the reptation analysis in advancing the idea that conformational dynamics strongly differentiate PU.1/DNA and Ets-1/DNA complexes. Both approaches show a dynamic coupling in the formation of site-specific complexes by PU.1 that is far less pronounced in the minimal ETS domain of Ets-1, and altogether undetectable when autoinhibitory helices are present. The amino acid diversity among ETS domains therefore encodes structurally homologous structures with divergent dynamic properties. Interestingly, solution NMR studies have revealed that elements N-terminal to the auto-inhibited helices, which are themselves intrinsically disordered (35–37), modify DNA binding through local interactions with the ETS domain. The present data indicate, however, that interactions involving only the auto-inhibitory helices do not significantly modify the global dynamics rooted in the minimal ETS domain of Ets-1.

### The heterogeneous dynamics of ETS/DNA complexes are not localized at the contact interface

Since the divergent dynamics between the ETS domains of PU.1 and Ets-1 are coupled to DNA site discrimination, we were interested to see if they are manifest locally at the protein/DNA interface. Specifically, if the ensemble of PU.1/DNA structures include highly transient interfacial interactions, their interfaces should be, on average, more solvent-exposed and susceptible to small chemical probes relative to Ets-1/DNA complexes. Dimethyl sulfate (DMS), which selectively methylates N^7^ positions of guanines via the DNA major groove, is well-suited to probe the interfacial accessibility at the 5′-GGAA-3′ consensus. Although DMS footprinting has been extensively used to map binding sites of individual ETS proteins, to our knowledge, it has not been used to compare DNA complexes of different ETS members.

**Figure 5.**
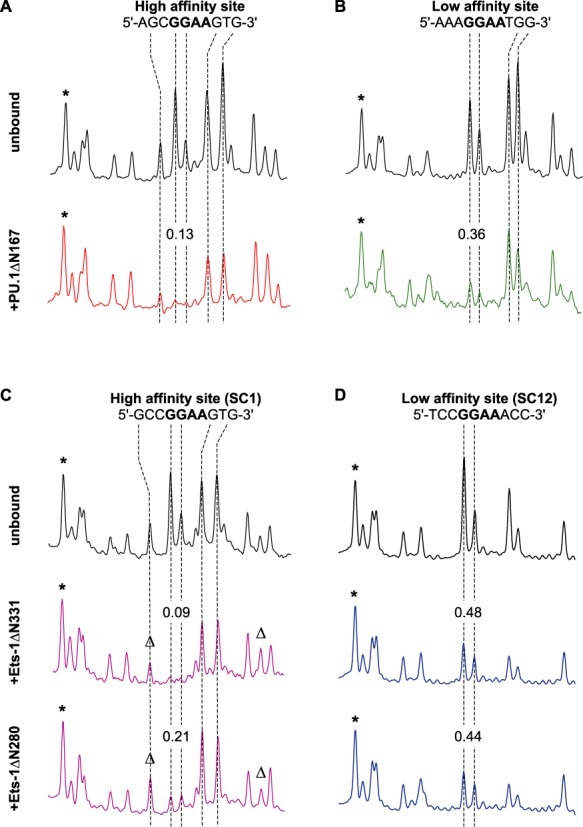
Differences in accessibility of the protein/DNA contact interface to dimethyl sulfate do not track the dynamic coupling to DNA site discrimination by PU.1 and Ets-1. DNA fragments harboring high- or low-affinity ETS binding sites, alone or incubated to equilibrium with saturating concentrations of PU.1ΔN167, Ets-1ΔN331 or Ets-1ΔN280, were subjected to limited methylation with DMS and subsequent strand scission with piperidine. Shown are capillary electropherograms excerpted around the ETS binding sites of the 5′-GGAA-3′ strand, normalized to the intensity of a distal control peak (marked with *). Additional peaks whose areas differ by >20% between the bound and unbound states are marked with Δ. Reactivity to DMS of the two consensus guanines indicates accessibility of the ETS/DNA core interface, while the major grooves of flanking guanines face outward from the protein. Numbers indicate the fractional integrated peak area for the two core guanines relative to that of the control peak, with a precision of ±0.1 in all cases.

We saturated fluorescently-labeled DNA fragments harboring various ETS binding sites with their protein targets at 10-fold or higher concentrations (0.1–10 μM) over their respective equilibrium dissociation constants, before reaction with DMS. Cleaved fragments were resolved by capillary electrophoresis (Figure 5). To verify the formation of site-specific complexes, each mixture was separately probed with DNase I. Hypersensitivity to DNase I, detected in the 5′-TTCC-3′ strand, is diagnostic for site-specific ETS/DNA complexes (38) in which the protein widens the DNA minor groove at the core consensus, but is absent in nonspecific binding (14). Since all complexes are DNase I-hypersensitive (Supplementary Figure S3), DMS sensitivity represents the kinetic accessibility of the major groove in each site-specific ETS/DNA complex.

To quantify the reactivity to DMS and to account for differences in recovery from purification steps, integrated peak areas corresponding to the two consensus guanines were normalized to a well-defined peak outside the binding interface (marked with ‘*’ in Figure 5) (39,40). We observed that the high-affinity interface for PU.1ΔN167 is essentially as well protected against DMS modification as Ets-1ΔN331 (∼90% relative to unbound). Thus, the broader ensemble of high-affinity PU.1/DNA structures does not significantly differ in interfacial accessibility from their Ets-1/DNA counterparts. In contrast, the interface of the low-affinity Ets-1ΔN331/SC12 complex is not only more accessible to DMS than its high-affinity counterpart, but unexpectedly also relative to the low-affinity PU.1/DNA complex. With respect to Ets-1 autoinhibition, the high-affinity complex formed by Ets-1ΔN280 is significantly more sensitive to DMS than Ets-1ΔN331. In addition, guanines flanking the core consensus, which are completely solvent-exposed at the major groove, are differentially methylated (marked ‘Δ’) between the autoinhibited and minimal Ets-1/DNA complexes. Finally, the high- and low-affinity Ets-1ΔN280 complexes differ in DMS sensitivity by a much smaller margin than their minimal Ets-1 domain counterparts (Ets-1ΔN331). Thus, interfacial perturbations induced by Ets-1ΔN331 are abrogated by the auto-inhibitory helices, suggesting an allosteric effect of autoinhibition on the protein/DNA interface in solution.

In summary, accessibility of the contact interfaces in PU.1/DNA and Ets-1/DNA complexes to DMS does not track the heterogeneous dynamics captured by the circular permutation and DLS studies. We therefore infer that, although the dynamic differences between the two ETS domains are coupled to DNA site discrimination, they are not localized at their DNA contact interface. The robust protection of the high-affinity contact interface by PU.1, despite its global dynamics, is in agreement with our earlier observation that PU.1 forms a kinetically persistent high-affinity complex (15,16). In addition, we have observed that the high-affinity PU.1/DNA complex is quantitatively destabilized by osmotic stress to an extent ∼10-fold greater than afforded by the sequestration of water molecules at the contact interface alone (14). If the dynamic changes in site discrimination by PU.1 are delocalized among elements distal to the DNA contact interface, the attendant changes in preferential hydration at mobile accessible surfaces may account for the magnitude of PU.1's osmotic sensitivity. Accordingly, the weak dynamic coupling by Ets-1 in DNA binding complements the osmotic insensitivity of Ets-1/DNA complexes (15). Thus, PU.1's osmotic sensitivity appears to represent the emergent property of an induced-fit mechanism involving the direct participation of water molecules in site recognition.

### Dynamics, heterogeneity and DNA site selectivity

Increasing awareness that co-expressing ETS members regulate distinct genetic networks *in vivo* (41–45) highlights the need for understanding how structurally homologous ETS proteins resolve their overlapping DNA sequence preferences. The current paradigm of site discrimination by ETS proteins posits a ‘direct’ readout of specific protein-DNA contacts at the 5′-GGA(A/T)-3′ core consensus and an ‘indirect’ readout of sequence-dependent backbone properties at the flanking bases that together define the broad sequence variation in ETS binding sites (46). While a dynamic component may be implied in this model, the present data show that dynamics are explicitly coupled to DNA site discrimination by PU.1, but not its structural homolog Ets-1.

**Figure 6.**
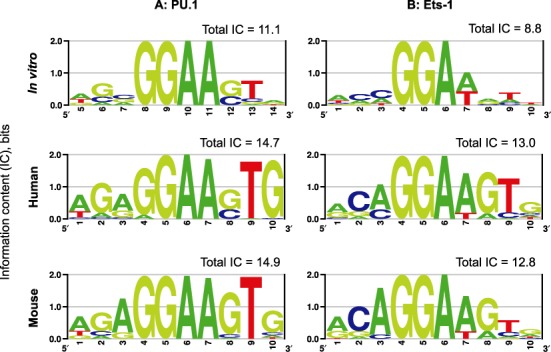
PU.1 extracts higher DNA site specificity than Ets-1 *in vivo* and *in vitro*. DNA site preferences *in vitro* by Ray-Gallet *et al*. (48) for PU.1 and Woods *et al*. (49) for Ets-1 were chosen among alternatives (21,46) for their larger sample sizes and sequence spaces. ChIP-Seq data for genomic preferences *in vivo* were as curated by the HOMER Motif Database for murine PU.1 (4) and human Ets-1 (50), the JASPAR Database for murine Ets-1 (MA0098), and the human ENCODE Consortium for human PU.1 (51). The data were analyzed for their information content (IC) and presented as DNA logos (47). The height of each stack represents the IC for that position, and summed to give the total IC for the 10-bp binding site. Although the total ICs differ depending on the experimental context, each matched pair of binding motifs differs by ∼2 bits.

What is the functional significance of the differential dynamic coupling to DNA site discrimination by ETS homologs? Although PU.1 and Ets-1 share overlapping sequence preferences, binding motifs for PU.1 and Ets-1 *in vivo* and *in vitro* show clear though non-exclusive differences in the bases flanking the consensus as well as Ets-1's tolerance for T at 5′-GGA(A/T)-3′ (Figure 6). Notably, the two homologs’ preferences are conserved whether determined *in vivo* by ChIP-sequencing or under cell-free conditions by selection experiments, indicating that their sequence preferences are intrinsic to their corresponding ETS domains.

Relative to Ets-1, we propose that dynamic coupling in DNA discrimination affords PU.1 distinct and more stringent sequence selectivity. To assess the site stringency of PU.1 and Ets-1, we computed the information content of their binding motifs (47,48–51). Assuming all four bases are equiprobable in nonspecific binding, the information content (IC, in binary bits) at each position of a specific binding site ranges from 0 if all bases remain equiprobable (i.e. maximum informational entropy), to 2 if a single base is absolutely preferred. The total IC for a binding site is the sum of its positional ICs. Our analysis shows that, while the total ICs for *in vivo* binding motifs are higher than *in vitro* binding, the binding motifs for PU.1 are consistently associated with higher total ICs than their Ets-1 counterparts by ∼2 bits (Figure 6). Since the primary data were obtained independently using different technologies, it is highly improbable that this agreement represents experimental artefacts.

To extend the analysis, the difference in the total ICs between the sequence motifs for two proteins (A and B) is related to their energetics of site discrimination (52):

(2) \begin{equation*} \Delta {\rm (total \ IC)}_{{\rm A - B}} = \varepsilon _r \left( {\log _2 \frac{{K_{{\rm sp,A}} }}{{K_{{\rm ns,A}} }} - \log _2 \frac{{K_{{\rm sp,B}} }}{{K_{{\rm ns,B}} }}} \right) \end{equation*}

The parenthetic factor on the right side of Equation (2) represents the maximum information (in bits) that can be gained as the proteins transition from nonspecific to specific binding (characterized by the equilibrium binding constants *K*_ns_ and *K*_sp_), with a maximum efficiency of *ε*_r_ = ln 2 ≈ 0.7 under isothermal conditions (52). We used Equation (2) to compare PU.1 and Ets-1 using reported values of specific and nonspecific binding. Under physiological saline conditions (150 mM Na^+^), high-affinity binding by PU.1 and Ets-1 are similar (10^−10^ M) (15). However, nonspecific binding by PU.1 (10^−5^ M) (14) is significantly weaker than Ets-1 (10^−7^ M) (21,53), a phenomenon that may be demonstrated directly (Supplementary Figure S4). Equation (2) shows that PU.1 can extract >2 additional bits of sequence selectivity by suppressing non-specific binding relative to Ets-1 (Supplementary Table S2), in agreement with the difference in total IC based on binding motifs (cf. Figure 6). Such a correspondence assumes that the two proteins are optimized DNA discriminators, a supposition supported by the overwhelming sequence conservation of their orthologs among high-order metazoans (54).

Thus, evidence from experimental and theoretical approaches supports the notion that PU.1 is a more sequence-selective protein that Ets-1. This feature is intuitively consistent with PU.1's status as a pioneer transcription factor, a function not shared by Ets-1 (8). Our present data, which show that the two proteins also differ strongly in conformational dynamics and its coupling to DNA site discrimination, suggest dynamics as a key component in the DNA site selectivity of the two ETS homologs. In conclusion, the interplay between dynamics, preferential interactions, kinetic persistence, and sequence selection presents a promising line of investigation into the biophysical mechanism of DNA site discrimination among co-expressing ETS proteins, and ultimately, how they specifically regulate their target genes in key developmental programs such as hematopoiesis and neurogenesis.

## SUPPLEMENTARY DATA

Supplementary Data are available at NAR Online.

SUPPLEMENTARY DATA

## References

[1] Zaret K.S., Carroll J.S. (2011). Pioneer transcription factors: establishing competence for gene expression. Genes Dev..

[2] Iwafuchi-Doi M., Zaret K.S. (2014). Pioneer transcription factors in cell reprogramming. Genes Dev..

[3] Pham T.H., Benner C., Lichtinger M., Schwarzfischer L., Hu Y., Andreesen R., Chen W., Rehli M. (2012). Dynamic epigenetic enhancer signatures reveal key transcription factors associated with monocytic differentiation states. Blood.

[4] Heinz S., Benner C., Spann N., Bertolino E., Lin Y.C., Laslo P., Cheng J.X., Murre C., Singh H., Glass C.K. (2010). Simple combinations of lineage-determining transcription factors prime cis-regulatory elements required for macrophage and B cell identities. Mol. Cell.

[5] Pham T.H., Minderjahn J., Schmidl C., Hoffmeister H., Schmidhofer S., Chen W., Langst G., Benner C., Rehli M. (2013). Mechanisms of in vivo binding site selection of the hematopoietic master transcription factor PU.1. Nucleic Acids Res..

[6] Ghisletti S., Barozzi I., Mietton F., Polletti S., De Santa F., Venturini E., Gregory L., Lonie L., Chew A., Wei C.L. (2010). Identification and characterization of enhancers controlling the inflammatory gene expression program in macrophages. Immunity.

[7] Schonheit J., Kuhl C., Gebhardt M.L., Klett F.F., Riemke P., Scheller M., Huang G., Naumann R., Leutz A., Stocking C. (2013). PU.1 level-directed chromatin structure remodeling at the Irf8 gene drives dendritic cell commitment. Cell Rep..

[8] Sherwood R.I., Hashimoto T., O'Donnell C.W., Lewis S., Barkal A.A., van Hoff J.P., Karun V., Jaakkola T., Gifford D.K. (2014). Discovery of directional and nondirectional pioneer transcription factors by modeling DNase profile magnitude and shape. Nat. Biotechnol..

[9] Graves B.J., Petersen J.M. (1998). Specificity within the ets family of transcription factors. Adv. Cancer Res..

[10] Wei G.H., Badis G., Berger M.F., Kivioja T., Palin K., Enge M., Bonke M., Jolma A., Varjosalo M., Gehrke A.R. (2010). Genome-wide analysis of ETS-family DNA-binding *in vitro* and *in vivo*. EMBO J..

[11] Garvie C.W., Hagman J., Wolberger C. (2001). Structural studies of Ets-1/Pax5 complex formation on DNA. Mol. Cell.

[12] Kodandapani R., Pio F., Ni C.Z., Piccialli G., Klemsz M., McKercher S., Maki R.A., Ely K.R. (1996). A new pattern for helix-turn-helix recognition revealed by the PU.1 ETS-domain-DNA complex. Nature.

[13] Mosca R., Schneider T.R. (2008). RAPIDO: a web server for the alignment of protein structures in the presence of conformational changes. Nucleic Acids Res..

[14] Poon G.M.K. (2012). Sequence discrimination by DNA-binding domain of ETS family transcription factor PU.1 is linked to specific hydration of protein-DNA interface. J. Biol. Chem..

[15] Wang S., Linde M.H., Munde M., Carvalho V.D., Wilson W.D., Poon G.M. (2014). Mechanistic heterogeneity in site recognition by the structurally homologous DNA-binding domains of the ETS family transcription factors Ets-1 and PU.1. J. Biol. Chem..

[16] Munde M., Poon G.M., Wilson W.D. (2013). Probing the electrostatics and pharmacological modulation of sequence-specific binding by the DNA-binding domain of the ETS family transcription factor PU.1: a binding affinity and kinetics investigation. J. Mol. Biol..

[17] Hollenhorst P.C., McIntosh L.P., Graves B.J. (2011). Genomic and biochemical insights into the specificity of ETS transcription factors. Annu. Rev. Biochem..

[18] Poon G.M., Macgregor R.B. Jr (2004). A thermodynamic basis of DNA sequence selectivity by the ETS domain of murine PU.1. J. Mol. Biol..

[19] Jonsen M.D., Petersen J.M., Xu Q.P., Graves B.J. (1996). Characterization of the cooperative function of inhibitory sequences in Ets-1. Mol. Cell. Biol..

[20] Poon G.M., Macgregor R.B. Jr (2003). Base coupling in sequence-specific site recognition by the ETS domain of murine PU.1. J. Mol. Biol..

[21] Nye J.A., Petersen J.M., Gunther C.V., Jonsen M.D., Graves B.J. (1992). Interaction of murine ets-1 with GGA-binding sites establishes the ETS domain as a new DNA-binding motif. Genes Dev..

[22] Tataurov A.V., You Y., Owczarzy R. (2008). Predicting ultraviolet spectrum of single stranded and double stranded deoxyribonucleic acids. Biophys. Chem..

[23] Zwieb C., Adhya S., Leblanc B, Moss T (2009). DNA-Protein Interactions : Principles and Protocols.

[24] Wilson D.O., Johnson P., McCord B.R. (2001). Nonradiochemical DNase I footprinting by capillary electrophoresis. Electrophoresis.

[25] Mitra S., Shcherbakova I.V., Altman R.B., Brenowitz M., Laederach A. (2008). High-throughput single-nucleotide structural mapping by capillary automated footprinting analysis. Nucleic Acids Res..

[26] He G., Vasilieva E., Bashkin J.K., Dupureur C.M. (2013). Mapping small DNA ligand hydroxyl radical footprinting and affinity cleavage products for capillary electrophoresis. Anal. Biochem..

[27] Poon G.M.K. (2012). DNA binding regulates the self-association of the ETS domain of PU.1 in a sequence-dependent manner. Biochemistry.

[28] Benjamini Y., Hochberg Y. (1995). Controlling the false discovery rate: a practical and powerful approach to multiple testing. J. Roy. Stat. Soc. B. Met..

[29] Strauss-Soukup J.K., Maher L.J. 3rd (1997). Role of asymmetric phosphate neutralization in DNA bending by PU.1. J. Biol. Chem..

[30] Ferrari S., Harley V.R., Pontiggia A., Goodfellow P.N., Lovell-Badge R., Bianchi M.E. (1992). SRY, like HMG1, recognizes sharp angles in DNA. EMBO J..

[31] Lumpkin O.J., Zimm B.H. (1982). Mobility of DNA in gel electrophoresis. Biopolymers.

[32] Levene S., Zimm B. (1989). Understanding the anomalous electrophoresis of bent DNA molecules: a reptation model. Science.

[33] Jia X., Lee L.K., Light J., Palmer A.G. 3rd, Assa-Munt N. (1999). Backbone dynamics of a short PU.1 ETS domain. J. Mol. Biol..

[34] Petersen J.M., Skalicky J.J., Donaldson L.W., McIntosh L.P., Alber T., Graves B.J. (1995). Modulation of transcription factor Ets-1 DNA binding: DNA-induced unfolding of an alpha helix. Science.

[35] Desjardins G., Meeker C.A., Bhachech N., Currie S.L., Okon M., Graves B.J., McIntosh L.P. (2014). Synergy of aromatic residues and phosphoserines within the intrinsically disordered DNA-binding inhibitory elements of the Ets-1 transcription factor. Proc. Natl. Acad. Sci. U.S.A..

[36] Pufall M.A., Lee G.M., Nelson M.L., Kang H.-S., Velyvis A., Kay L.E., McIntosh L.P., Graves B.J. (2005). Variable control of Ets-1 DNA dinding by multiple phosphates in an unstructured region. Science.

[37] Lee G.M., Pufall M.A., Meeker C.A., Kang H.S., Graves B.J., McIntosh L.P. (2008). The affinity of Ets-1 for DNA is modulated by phosphorylation through transient interactions of an unstructured region. J. Mol. Biol..

[38] Graves B.J., Gillespie M.E., McIntosh L.P. (1996). DNA binding by the ETS domain. Nature.

[39] Bashkin J.K., Aston K., Ramos J.P., Koeller K.J., Nanjunda R., He G., Dupureur C.M., David Wilson W. (2013). Promoter scanning of the human COX-2 gene with 8-ring polyamides: unexpected weakening of polyamide-DNA binding and selectivity by replacing an internal N-Me-pyrrole with beta-alanine. Biochimie.

[40] He G., Vasilieva E., Harris G.D. Jr, Koeller K.J., Bashkin J.K., Dupureur C.M. (2014). Binding studies of a large antiviral polyamide to a natural HPV sequence. Biochimie.

[41] Ciau-Uitz A., Wang L., Patient R., Liu F. (2013). ETS transcription factors in hematopoietic stem cell development. Blood Cells Mol. Dis..

[42] Odrowaz Z., Sharrocks A.D. (2012). The ETS transcription factors ELK1 and GABPA regulate different gene networks to control MCF10A breast epithelial cell migration. PLoS One.

[43] Spooner C.J., Cheng J.X., Pujadas E., Laslo P., Singh H. (2009). A recurrent network involving the transcription factors PU.1 and Gfi1 orchestrates innate and adaptive immune cell fates. Immunity.

[44] Weigelt K., Lichtinger M., Rehli M., Langmann T. (2009). Transcriptomic profiling identifies a PU.1 regulatory network in macrophages. Biochem. Biophys. Res. Commun..

[45] Wontakal S.N., Guo X., Will B., Shi M., Raha D., Mahajan M.C., Weissman S., Snyder M., Steidl U., Zheng D. (2011). A large gene network in immature erythroid cells is controlled by the myeloid and B cell transcriptional regulator PU.1. PLoS Genet..

[46] Szymczyna B.R., Arrowsmith C.H. (2000). DNA binding specificity studies of four ETS proteins support an indirect read-out mechanism of protein-DNA recognition. J. Biol. Chem..

[47] Schneider T.D., Stormo G.D., Gold L., Ehrenfeucht A. (1986). Information content of binding sites on nucleotide sequences. J. Mol. Biol..

[48] Ray-Gallet D., Mao C., Tavitian A., Moreau-Gachelin F. (1995). DNA binding specificities of Spi-1/PU.1 and Spi-B transcription factors and identification of a Spi-1/Spi-B binding site in the c-fes/c-fps promoter. Oncogene.

[49] Woods D.B., Ghysdael J., Owen M.J. (1992). Identification of nucleotide preferences in DNA sequences recognised specifically by c-Ets-1 protein. Nucleic Acids Res..

[50] Hollenhorst P.C., Chandler K.J., Poulsen R.L., Johnson W.E., Speck N.A., Graves B.J. (2009). DNA specificity determinants associate with distinct transcription factor functions. PLoS Genet..

[51] Wang J., Zhuang J., Iyer S., Lin X., Whitfield T.W., Greven M.C., Pierce B.G., Dong X., Kundaje A., Cheng Y. (2012). Sequence features and chromatin structure around the genomic regions bound by 119 human transcription factors. Genome Res..

[52] Schneider T.D. (2010). 70% efficiency of bistate molecular machines explained by information theory, high dimensional geometry and evolutionary convergence. Nucleic Acids Res..

[53] Goetz T.L., Gu T.L., Speck N.A., Graves B.J. (2000). Auto-inhibition of Ets-1 is counteracted by DNA binding cooperativity with core-binding factor alpha2. Mol. Cell. Biol..

[54] Pió F., Kodandapani R., Ni C.Z., Shepard W., Klemsz M., McKercher S.R., Maki R.A., Ely K.R. (1996). New insights on DNA recognition by ets proteins from the crystal structure of the PU.1 ETS domain-DNA complex. J. Biol. Chem..

