# Supplementary material for: Heterogeneous dynamics in DNA site discrimination by the structurally homologous DNA-binding domains of ETS-family transcription factors
Source: Nucleic Acids Res. 2015 Mar 30;43(8):4322–31. doi: 10.1093/nar/gkv267 (PMC4417174; doi:10.1093/nar/gkv267)
Supplement: SUPPLEMENTARY DATA [file supp_43_8_4322__index.html]

Heterogeneous dynamics in DNA site discrimination by the structurally homologous DNA-binding domains of ETS-family transcription factors — SUPPLEMENTARY DATA 

# Heterogeneous dynamics in DNA site discrimination by the structurally homologous DNA-binding domains of ETS-family transcription factors

## SUPPLEMENTARY DATA

**Files in this Data Supplement:**

- SUPPLEMENTARY DATA
