# Supplementary material for: Heterogeneous dynamics in DNA site discrimination by the structurally homologous DNA-binding domains of ETS-family transcription factors
Source: Nucleic Acids Res. 2015 Mar 30;43(8):4322–31. doi: 10.1093/nar/gkv267 (PMC4417174; doi:10.1093/nar/gkv267)
Supplement: SUPPLEMENTARY DATA [file supp_gkv267_nar-03669-m-2014-File008.pdf]

SUPPLEMENTARY MATERIALS

for

Heterogeneous dynamics in DNA site discrimination by the structurally homologous DNA-binding domains of ETS-family transcription factors

Gaofei He,[1] Ana Tolic,[2] James K. Bashkin,[1] and Gregory M. K. Poon [2,3,*]

March 4, 2015

[1] Department of Chemistry and Biochemistry and Center for Nanoscience, University of Missouri – St. Louis, St. Louis, MO 63121, USA

[2] College of Pharmacy, Washington State University, Spokane, WA 99210, USA

[3] Current address: Department of Chemistry and the Byrdine F. Lewis School of Nursing and Health Professions, Georgia State University, Georgia, GA 30303, USA

* Address correspondence to G.M.K. Poon (gpoon@gsu.edu).

**SUPPLEMENT INDEX**	**PAGE**
Supplementary Methods	2 to 5
Summary of Supplementary Figures	6
Supplementary Tables and Figures	7 to 13
References for Supplementary Materials	14

SUPPLEMENTARY METHODS

SM1. DNA site circular permutation: molecular cloning. Oligos encoding high- and low-affinity binding sites for PU.1 and Ets-1 were synthesized in the format:

5'- CTAGAGAAATANNN**GGAA**NNNAAACCAG -3'
3'- TCTTTATNNN**CCTT**NNNTTTGGTCAGCT -5'

ETS member	DNA site	Sequence	Dissociation constant, M
PU.1	High-affinity	AGC**GGAA**GTG	7×10^{-10}
	Low-affinity	AAA**GGAA**TGG	2×10^{-7}
Ets-1	High-affinity (SC1)	GCC**GGAA**GTG	3×10^{-10}
	Low-affinity (SC12)	TCC**GGAA**ACC	6×10^{-9}

The dissociation constants listed refer to values determined under physiologically saline conditions (150 mM Na^+) at 25°C (1). Note: SC12 is the weakest GGAA-bearing site identified from an *in vitro* SELEX screen (2). The duplex oligos were cloned between the XbaI (T↓CTAGA) and SalI sites (G↓TCGAC) in pBend5 vector (ATCC; 3) that has been propagated in methylation-deficient GM2929 *E. coli* (a kind gift from Dr. Martin Marius, University of Massachusetts Medical School). Plasmids were first transformed into DH5α *E. coli*, verified by sequencing, and subsequently maintained in GM2929 cells. Mini-preps were restriction-digested, in separate reactions, to yield eleven circularly permuted, 143-bp fragments (**Figure SM1**). Each fragment was purified from 3% agarose gels with spin columns (Thermo Scientific) and used as described in *Materials and Methods* in the main text.

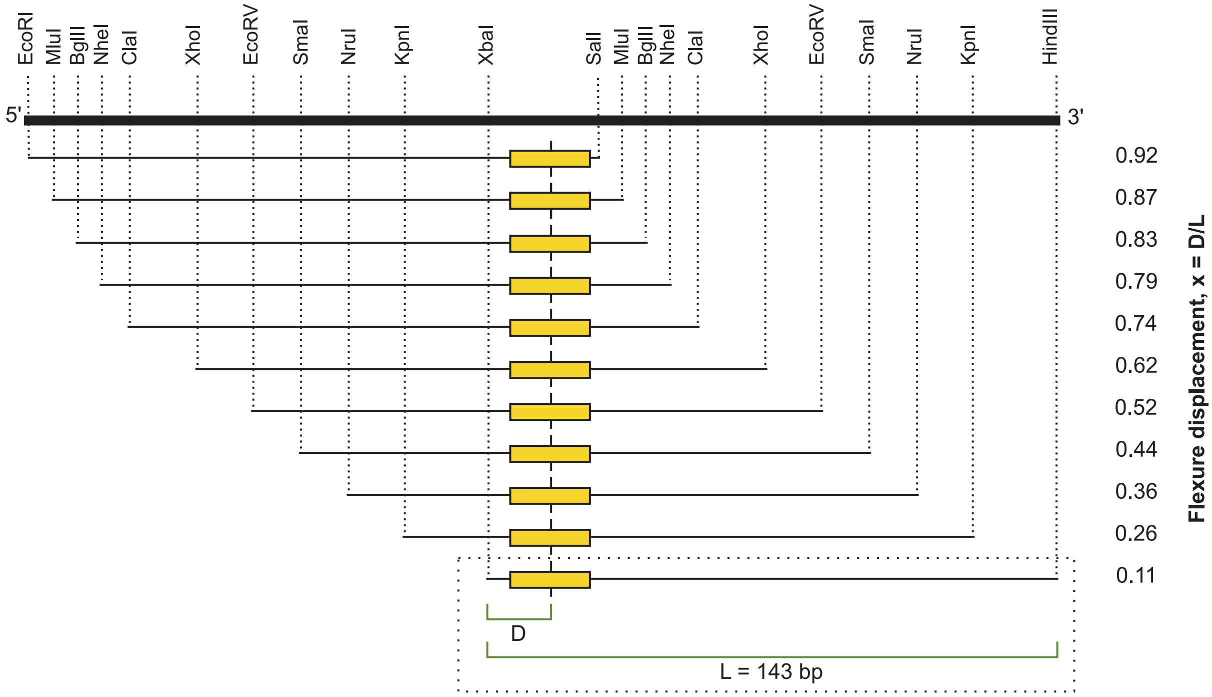

Figure SM1. Schematic of the circularly permutated DNA fragments used to probe the electrophoretic properties of site-specific complexes formed by ETS domains. ETS binding sites are shown as yellow boxes.

SM2. Electrophoretic measurements of mobility shift data. Dried polyacrylamide gels were digitized by phosphorimagery at 100 μm spatial resolution and visualized with ImageJ software (v1.8, NIH). One-dimensional Intensity traces for each lane were generated by integrating the intensity orthogonally to the direction of electrophoresis, as illustrated for PU.1ΔN167 bound at flexure displacement x = 0.91 (**Figure SM2, Panel A**). The gels were run to maximize the dispersion of the bound species without running the unbound (faster) species off the gel, which is 10 cm = 100 mm (corresponding to 1,000 pixels) in length from well to bottom of gel. Under these conditions, the bound bands traveled absolute distances of approximately ~9 cm, corresponding to a distance of 5 to 8 cm for the bound species, depending on the protein and flexure displacement of the binding site within the DNA fragment.

Peak positions for the unbound and protein-bound bands were quantitatively determined by standard first derivative analysis (**Figure SM2, Panel B**) (4). Specifically, for each lane, a first derivative trace was constructed by numerical differentiation of the intensity trace I with respect to position z, without smoothing, by computing the central derivative at each point (indexed by pixel count i):

$$\frac{dI(z_i)}{dz_i} = \frac{1}{2}\left(\frac{I(z_{i+1}) - I(z_i)}{z_{i+1} - z_i} + \frac{I(z_i) - I(z_{i-1})}{z_i - z_{i-1}} \right) . \qquad (1)$$

Each peak z_p is taken as the point z_i at which the derivative crosses from positive to negative i.e., $dI/dz > 0$ at z_{i-1} and $dI/dz < 0$ at z_i (**Figure SM2, Panel C**). For each flexure displacement x, relative mobility (R_f) is defined as

$$R_f(x) \equiv \frac{z_{p,b}(x)}{z_{p,u}(x)} \ . \qquad (2)$$

The precision of a single determination of R_f is propagated from the precisions of the constituent peak positions, Δz_p, following the definition of R_f [Eq. (2)], by:

$$\Delta R_f = \Delta\left(\frac{z_{p,b}}{z_{p,u}} \right) = \frac{z_{p,b}}{z_{p,u}} \sqrt{\left(\frac{\Delta z_{p,b}}{z_{p,b}} \right)^2 + \left(\frac{\Delta z_{p,u}}{z_{p,u}} \right)^2} \ . \qquad (3)$$

Since $\Delta z_p = \pm 1$ pixel (c.f. **Figure SM2, Panel C**), $|\Delta(z_p)/z_p| \leq 0.005$ and $0.6 < R_f < 0.9$ (c.f. Figure 2 in the main text), an analytical resolution of $|\Delta R_f| \leq 0.005$ is routinely possible. **In Figures 3D to F in the main text, each R_f is plotted as the mean value of quadruplicate samples ± S.E., and tabulated in Table S1.**

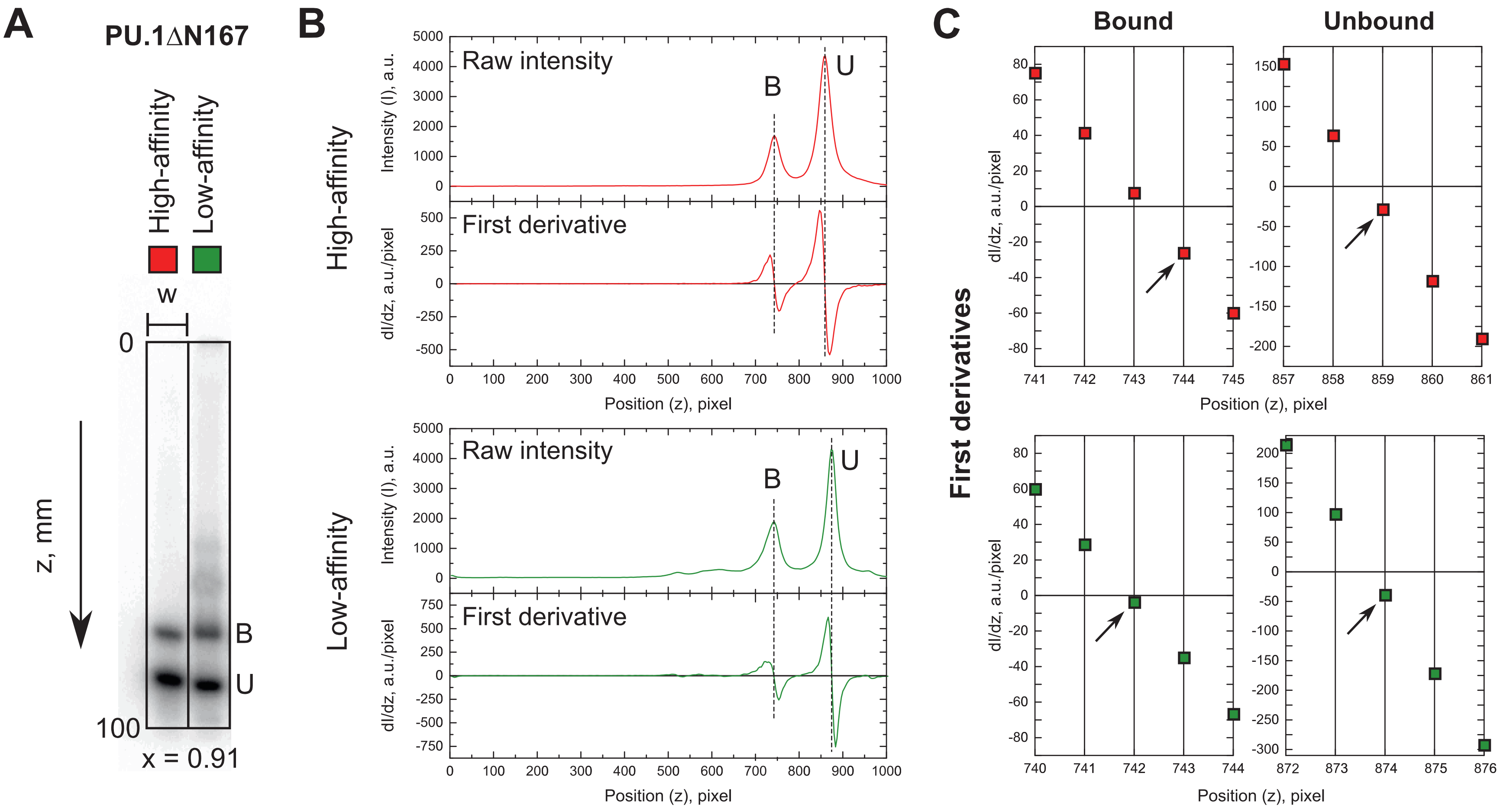

Figure SM2. Electrophoretic measurement of mobility shift by ETS-bound sequence-specific sites. The procedure is illustrated for one sample of PU.1ΔN167 boudn at flexure displacement x = 0.91 (c.f. Figure 2A in the main text). (**A**), Digitized image of the resolved free and ETS-bound DNA bands in native polyacrylamide gel at 100 μm resolution. For each lane, the area used for generating intensity traces are marked by open boxes, where the top (z = 0) and bottom (z = 100 mm, or 1,000 pixel counts) correspond to the bottom of the loading well and the end of the gel, respectively. The intensity bounded within each box is integrated across the width of the lane (w) to generate a one-dimensional intensity trace, I(z), as shown in **B**. Note the use of pixels as the unit in the abscissa; each pixel represents an independent instrumental measurement of intensity at that spatial coordinate. A first-derivative trace, dI/dz, is then generated by numerical differentiation with respect to z, using Eq. (1), to facilitate the measurement of peak positions (**C**), which are defined as the point z_i at which the derivative crosses from positive to negative i.e., $dI/dz > 0$ at z_{i-1} and $dI/dz < 0$ at z_i (marked with arrows). In this example, the relative mobilities, defined by the ratio of the bound to unbound peak position [Eq. (2)] are $R_f = 0.866$ for the high-affinity for PU.1ΔN167 complex and 0.849 for the low-affinity complex. It can be seen that the resolution in peak position is ±1 pixel, and the corresponding resolution in R_f is therefore ±0.005 or better.

SM3. Model-dependent analysis of circular permutation data. Our analysis is based on the work of Bianchi and coworkers (5) who modeled the electrophoretic mobilities of DNA fragments harboring circularly permuted DNA sites. In brief, the model combines the Lumpkin-Zimm reptation theory (6,7) with a geometric construct to give a quadratic relationship between the relative mobility of a DNA fragment and the flexure displacement (x) in the bound state:

$$R_f = \varphi x^2 - \varphi x + K\,, \qquad (4)$$

where

$$\varphi = 2K(1+\cos\gamma)$$

$$K = \frac{Q_b}{\zeta_b}\frac{\zeta_u}{Q_u}$$

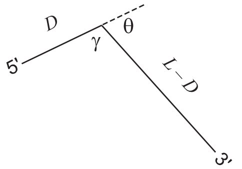

Q and ξ represent the total effective charge and frictional constant, respectively, of the unbound (u) and protein-bound (b) DNA fragment. The model assumes the protein-induced curvature as an oriented point-kink, which is appropriate given the short span of the ETS binding site (10 bp) relative to the length of the DNA fragment (143 bp) (c.f. Figure 2 in Wu et al., 2001; 8). As detailed in the main text, the key utility of Eq. (4) is that it parameterizes bend-related (θ = 180° – γ) and non-bend-related (K, a dimensionless quantity) determinants of electrophoretic mobility, thus enabling a direct comparison of complexes formed by different binding sites (e.g. high- and low-affinity) with the same protein.

Geometrically, Eq. (4) is a convex parabola centered at x = 0.5, the flexure displacement that induces maximal deviation from linearity in the DNA fragment. To estimate θ and K for each complex, Eq. (4) was used to fit mean values of R_f from quadruplicate independent measurements (tabulated in **Table S1**) as a function of x. Least-square fits and 95% confidence bands were plotted with the mean values of R_f together with in Figures 3D to F in the main text. **Note that R_f(x = 0) = R_f(x = 1) = K independently of γ; the functional value at x = 0 and x = 1 therefore provides a direct assessment of the non-bend-related parameters from a fit of Eq. (4) to the relative mobility data.**

SUMMARY OF SUPPLEMENTARY TABLES AND FIGURES

**Table S1**	Relative electrophoretic mobility measurements of PU.1 and Ets-1 bound to circularly permuted DNA sites.
**Table S2**	Model-dependent analysis of electrophoretic mobilities of PU.1 and Ets-1 bound to circularly permuted DNA sites.
**Table S3**	Information-based analysis of DNA site discrimination by PU.1 and Ets-1.
**Figure S1**	Electrophoretic analysis of purified ETS domains.
**Figure S2**	Dynamic light scattering (DLS) profiles of unbound DNA sites.
**Figure S3**	Sensitivity of ETS-bound DNA to DNase I confirms canonical sequence-specific ETS/DNA complexes
**Figure S4**	Probing the relative stringency of DNA site selection by PU.1 and Ets-1.

	Relative mobility (R_f)								
	PU.1			Ets-1Δ311			Ets-1Δ280		
**x**	**High-affinity**	**Low-affinity**	**p***	**High-affinity**	**Low-affinity**	**p***	**High-affinity**	**Low-affinity**	**p***
**0.11**	0.868 ± 0.003	0.846 ± 0.004	0.003	0.860 ± 0.002	0.868 ± 0.003	0.086	0.690 ± 0.005	0.692 ± 0.004	0.715
**0.26**	0.829 ± 0.002	0.822 ± 0.004	0.188	0.806 ± 0.001	0.812 ± 0.003	0.073	0.629 ± 0.004	0.635 ± 0.002	0.205
**0.36**	0.811 ± 0.004	0.815 ± 0.001	0.265	0.798 ± 0.003	0.808 ± 0.001	0.010	0.608 ± 0.003	0.608 ± 0.002	0.980
**0.44**	0.803 ± 0.002	0.808 ± 0.002	0.072	0.800 ± 0.003	0.804 ± 0.003	0.335	0.604 ± 0.004	0.606 ± 0.005	0.670
**0.52**	0.804 ± 0.003	0.803 ± 0.002	0.918	0.798 ± 0.002	0.803 ± 0.004	0.349	0.606 ± 0.002	0.602 ± 0.003	0.371
**0.62**	0.803 ± 0.001	0.805 ± 0.002	0.387	0.797 ± 0.002	0.803 ± 0.002	0.060	0.612 ± 0.002	0.610 ± 0.003	0.523
**0.74**	0.828 ± 0.002	0.823 ± 0.002	0.118	0.811 ± 0.003	0.816 ± 0.002	0.192	0.632 ± 0.002	0.626 ± 0.004	0.201
**0.79**	0.836 ± 0.002	0.824 ± 0.002	0.005	0.815 ± 0.002	0.821 ± 0.001	0.068	0.634 ± 0.004	0.635 ± 0.004	0.838
**0.83**	0.848 ± 0.002	0.836 ± 0.001	0.001	0.818 ± 0.004	0.827 ± 0.004	0.147	0.645 ± 0.005	0.643 ± 0.005	0.784
**0.87**	0.859 ± 0.001	0.845 ± 0.003	0.007	0.803 ± 0.003	0.839 ± 0.004	0.109	0.664 ± 0.001	0.670 ± 0.005	0.280
**0.92**	0.867 ± 0.001	0.853 ± 0.002	0.0004	0.842 ± 0.004	0.852 ± 0.002	0.064	0.689 ± 0.003	0.688 ± 0.002	0.753
		**FDR-adjusted p-value**	0.022			— **			— **

* Cells in orange highlight indicate significant difference between the high- and low-affinity complexes by *t* test alone ($p < 0.05$), and yellow highlights indicate significance following a false discovery rate (FDR) adjustment at 5%.
** No statistically significant differences following FDR adjustment.

Table S1

Relative electrophoretic mobility measurements of PU.1 and Ets-1 bound to circularly permuted DNA sites. Relative mobilities of ETS-bound DNA at different flexure displacements were determined as described in SM2 and tabulated as the mean ± S.E. of quadruplicate experiments. The experimental variation among replicas is comparable to the analytical precision of individual measurements (±≤0.005, c.f. **Figure SM2**). For each protein, adjustment for false discovery following multiple *t*-test comparisons of the high- and low-affinity complexes of each protein was computed from raw p values as described by Benjamini and Hochberg (9). After controlling for false discovery, only the complexes of PU.1 with high- and low-affinity terminal binding sites exhibit significantly different mobilities (adjusted $p < 0.022$; yellow highlights), while complexes of Ets-1 exhibit no significant differences at any flexure displacements.

ETS domain	DNA site	Θ, °	K*	p**
PU.1ΔN167	High-affinity	39.3 (37.6, 40.9)	0.904 (0.897, 0.911)	< 0.00001
	Low-affinity	32.6 (30.4, 34.6)	0.874 (0.867, 0.881)	
Ets-1ΔN331	High-affinity (SC1)	34.3 (30.3, 37.8)	0.869 (0.857, 0.882)	0.15
	Low-affinity (SC12)	35.3 (31.7, 38.5)	0.880 (0.868, 0.892)	
Ets-1ΔN280	High-affinity (SC1)	49.2 (46.9, 51.3)	0.726 (0.717, 0.735)	0.98
	Low-affinity (SC12)			

* K is a dimensionless quantity [c.f., Eq. (4)].
** p value as computed from a Fisher's *F* test on residual sums of squares.

Table S2
Model-dependent analysis of electrophoretic mobilities of PU.1 and Ets-1 bound to circularly permuted DNA sites. For each complex, Eq. (4) was used to fit the relative mobility data shown in Table S1 as a function of flexure displacement in order to estimate the bending angle (θ, in degrees) and the dimensionless non-bend related parameter K [c.f., Eq. (4)]. Parentheses indicate 95% joint confidence limits computed by the *F* test method for joint parameters. Differences between the fits of the data for high- and low-affinity complexes of each protein were tested by Fisher's *F* test on residual sum of squares:

$$F = \frac{(RSS_{\text{comb}} - RSS_{\text{sep}})/(df_{\text{comb}} - df_{\text{sep}})}{RSS_{\text{sep}}/df_{\text{sep}}} \tag{5}$$

Where RSS_{comb} and df_{comb} are the residual sum of squares and degrees of freedom from a fit of the two datasets combined and $RSS_{sep} = RSS_{high\text{-}affinity} + RSS_{low\text{-}affinity}$ and $df_{sep} = df_{high\text{-}affinity} + df_{low\text{-}affinity}$. At a significance level of p = 0.05, the model clearly distinguishes the high- and low-affinity complexes of PU.1ΔN167 ($p = 2 \times 10^{-6}$), but not for Ets-1ΔN331 and Ets-1ΔN280. In the case of Ets-1ΔN280, a global fit of the high- and low-affinity data is shown.

Protein	Dissociation constant, M		Ref.	R_{energy}, bits	Max. total IC, bits
	Specific ($K_{d,sp}$)	Nonspecific ($K_{d,ns}$)			
PU.1	7×10^{-10}	1×10^{-5}	(1,10)	13.8	9.7
Ets-1	3×10^{-10}	4×10^{-7}	(1,2,11)	10.5	7.3
					Δ(total IC) = 2.4

Table S3
Information-based analysis of DNA site discrimination by PU.1 and Ets-1. The information equivalent of the energetics for the nonspecific-to-specific transition by PU.1 and Ets-1 (R_{energy}, in binary bits) is computed via $\log_2(K_{d,ns}/K_{d,sp})$ using reported values for the equilibrium dissociation constants for high-affinity specific and nonspecific DNA binding by the two proteins. The extractable total IC for each protein is the fractional R_{energy} scaled by the theoretical maximum isothermal efficiency $\varepsilon_r = \ln 2 \approx 0.7$ (12). Thus, by suppressing nonspecific binding, PU.1 can extract over 2 additional bits of total IC relative to Ets-1. These steps are captured in Eq. (2) in the main text.

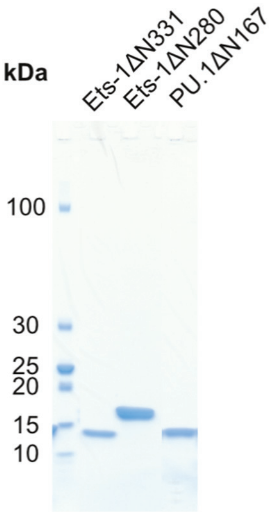

Figure S1
Electrophoretic analysis of purified ETS domains. Recombinant ETS domains of Ets-1 and PU.1 were routinely purified from *E. coli* under native conditions as previously described (1,10) and outlined in *Materials and Methods*. Briefly, 6xHis-tagged protein was extracted on Co-NTA resin, eluted, treated with thrombin to cleave the C-terminal fusion tag, and polished by size exclusion on a Superdex 75 10/300 column (GE Healthcare). Approximately 1 µg of protein was resolved by denaturing SDS-PAGE under reducing conditions, and stained with Coomassie Blue. No qualitative heterogeneity was observed. ETS domains are strongly basic proteins which migrate more slowly than expected based on molecular weight alone.

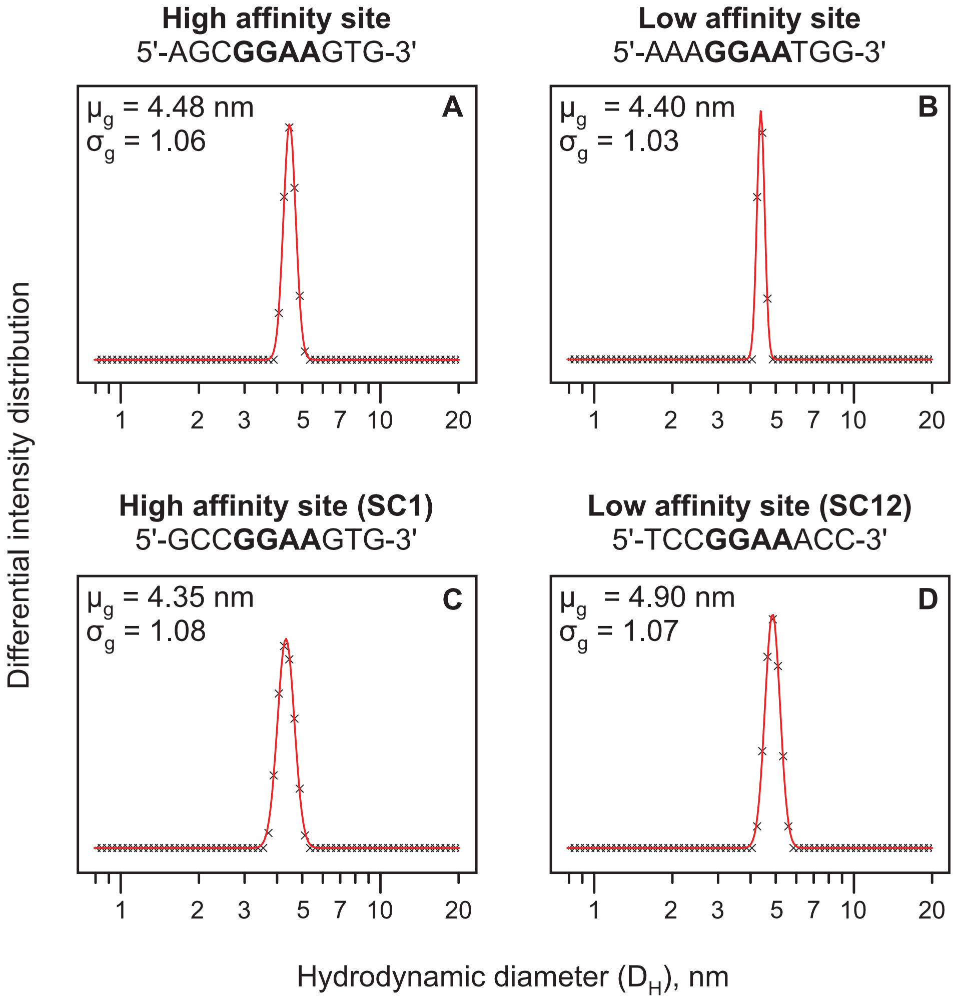

Figure S2

Dynamic light scattering (DLS) profiles of unbound DNA sites. DNA duplexes (23 bp, 200 μM) harboring the high- or low-affinity sequence-specific sites for PU.1 and Ets-1 were analyzed in free solution by DLS, as described in *Materials and Methods*, exactly as for the unbound and DNA duplex-bound proteins. Note that the DNA used in the DLS experiments are well below the persistence length for duplex DNA under ambient conditions (~150 bp) and tumble in solution effectively as rods. The hydrodynamic sizes detected by DLS therefore account for this highly non-spherical geometry as well as all associated counter-ions and hydration water. Accordingly, the oriented docking and redistribution of counter-ions plus water molecules that attend site-specific binding for both protein and DNA lead to non-additive changes (including reductions) in hydrodynamic diameter for the complex (c.f. Figure 4 in the main text).

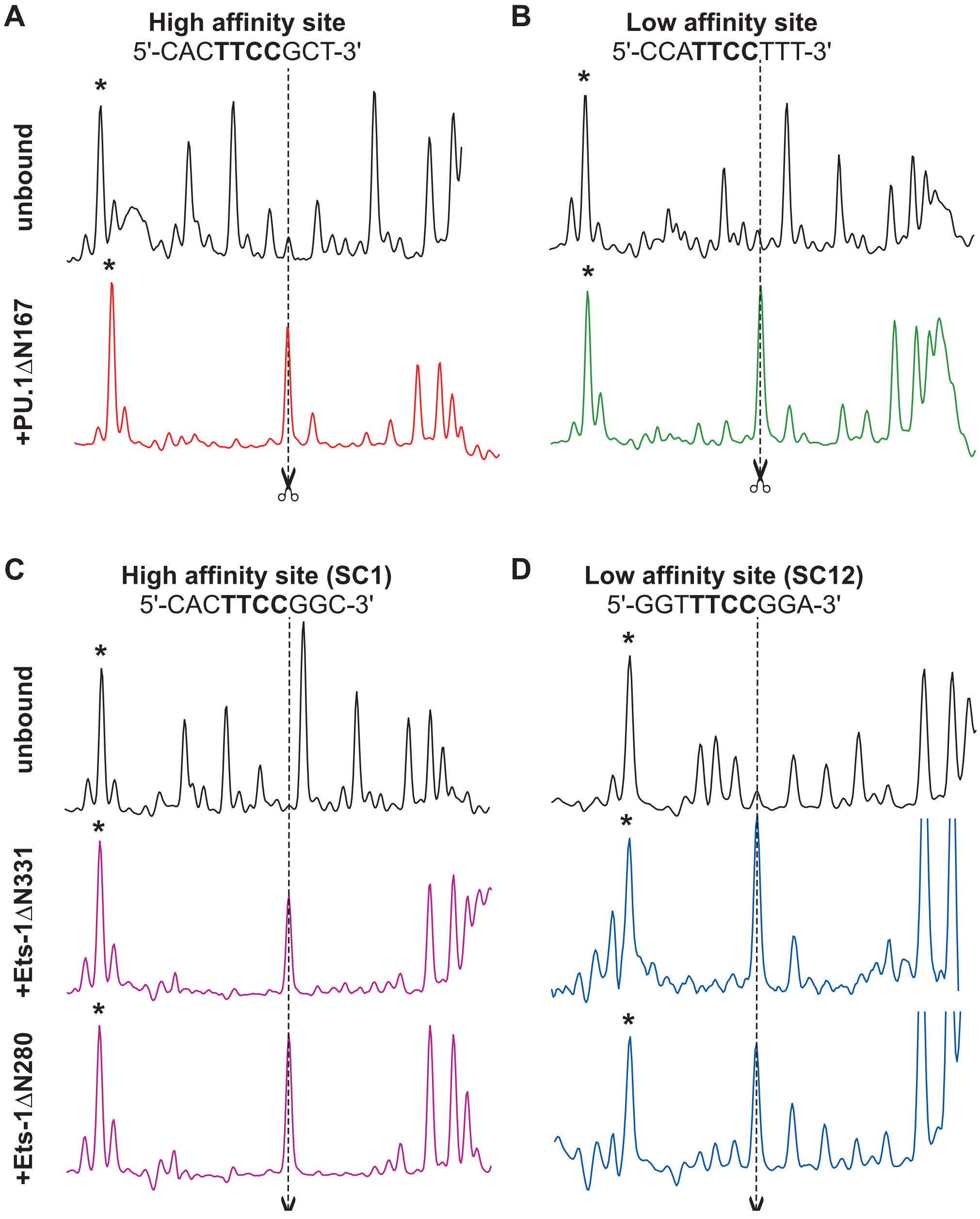

Figure S3
Sensitivity of ETS-bound DNA to DNase I confirms canonical sequence-specific ETS/DNA complexes. DNA fragments harboring high- or low-affinity ETS binding sites alone or incubated to equilibrium with saturating concentrations of PU.1ΔN167, Ets-1ΔN331, or Ets-1ΔN280, were subject to limited digestion with DNase I. Shown are capillary electropherograms excerpted around the ETS binding sites of the "reverse" (5'-TTCC-3') strand, normalized to the intensity of a distal control peak (marked with *). The presence of a DNase I-hypersensitive peak only at the indicated position is a diagnostic hallmark of all site-specific PU.1 and Ets-1/DNA complexes (13), due to protein-induced widening of the minor groove at the core consensus. Nonspecific complexes are characteristically not hypersensitive to DNase I (10). Unbound DNA is in black, ETS-bound DNA is in red (PU.1ΔN167, high-affinity), green (PU.1ΔN167, low-affinity), magenta (Ets-1, high-affinity), and blue (Ets-1, low-affinity).

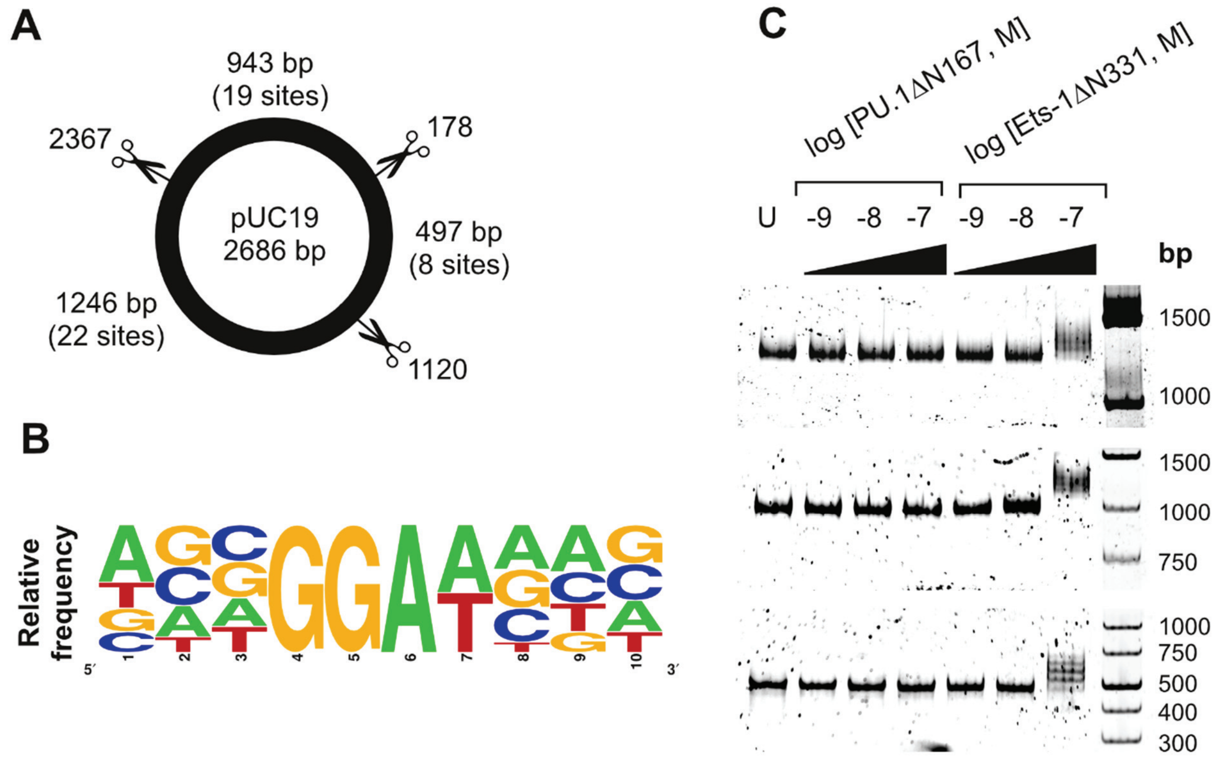

Figure S4

Probing the relative stringency of DNA site selection by PU.1 and Ets-1. We used pUC19, a mixed-sequence plasmid, to illustrate the relative stringency of sequence-specific site selection by PU.1 and Ets-1. pUC19 harbors 49 randomly-distributed ETS binding sites bearing a 5'-GGA(A/T)-3' core consensus. **A**, The plasmid, propagated in methylation-deficient GM2929 *E. coli*, was digested with ApaLI (G↓TGCAC) to generate three distinct fragments. **B**, Sequence logo for the set of 49 sites, with the base occurrences shown as relative frequencies at each position. **C**, Each gel-purified fragment was titrated with recombinant PU.1ΔN167 or Ets-1ΔN331 as shown (U = unbound). Each sample was separately resolved by electrophoretic mobility shift with a molecular weight ladder and stained with SYBR Gold (Life Technologies).

REFERENCES FOR SUPPLEMENTARY MATERIALS

1. Wang, S., Linde, M.H., Munde, M., Carvalho, V.D., Wilson, W.D. and Poon, G.M. (2014) Mechanistic heterogeneity in site recognition by the structurally homologous DNA-binding domains of the ETS family transcription factors Ets-1 and PU.1. *J Biol Chem*, **289**, 21605-21616.
2. Nye, J.A., Petersen, J.M., Gunther, C.V., Jonsen, M.D. and Graves, B.J. (1992) Interaction of murine ets-1 with GGA-binding sites establishes the ETS domain as a new DNA-binding motif. *Genes Dev*, **6**, 975-990.
3. Zwieb, C. and Adhya, S. (2009) In Leblanc, B. and Moss, T. (eds.). Humana Press, Vol. 543, pp. 547-562.
4. Brouwer, G. and Jansen, J.A.J. (1973) Deconvolution method for identification of peaks in digitized spectra. *Analytical Chemistry*, **45**, 2239-2247.
5. Ferrari, S., Harley, V.R., Pontiggia, A., Goodfellow, P.N., Lovell-Badge, R. and Bianchi, M.E. (1992) SRY, like HMG1, recognizes sharp angles in DNA. *EMBO J*, **11**, 4497-4506.
6. Lumpkin, O.J. and Zimm, B.H. (1982) Mobility of DNA in gel electrophoresis. *Biopolymers*, **21**, 2315-2316.
7. Levene, S. and Zimm, B. (1989) Understanding the anomalous electrophoresis of bent DNA molecules: a reptation model. *Science*, **245**, 396-399.
8. Wu, J., Parkhurst, K.M., Powell, R.M., Brenowitz, M. and Parkhurst, L.J. (2001) DNA bends in TATA-binding protein-TATA complexes in solution are DNA sequence-dependent. *J Biol Chem*, **276**, 14614-14622.
9. Benjamini, Y. and Hochberg, Y. (1995) Controlling the False Discovery Rate: A Practical and Powerful Approach to Multiple Testing. *J Roy Stat Soc B Met*, **57**, 289-300.
10. Poon, G.M.K. (2012) Sequence Discrimination by DNA-binding Domain of ETS Family Transcription Factor PU.1 Is Linked to Specific Hydration of Protein-DNA Interface. *J Biol Chem*, **287**, 18297-18307.
11. Goetz, T.L., Gu, T.L., Speck, N.A. and Graves, B.J. (2000) Auto-inhibition of Ets-1 is counteracted by DNA binding cooperativity with core-binding factor alpha2. *Mol Cell Biol*, **20**, 81-90.
12. Schneider, T.D. (2010) 70% efficiency of bistate molecular machines explained by information theory, high dimensional geometry and evolutionary convergence. *Nucleic Acids Res*, **38**, 5995-6006.
13. Graves, B.J., Gillespie, M.E. and McIntosh, L.P. (1996) DNA binding by the ETS domain. *Nature*, **384**, 322.
